# Association of War Zone–Related Stress With Alterations in Limbic Gray Matter Microstructure

**DOI:** 10.1001/jamanetworkopen.2022.31891

**Published:** 2022-09-16

**Authors:** Elisabeth Kaufmann, Philine Rojczyk, Valerie J. Sydnor, Jeffrey P. Guenette, Yorghos Tripodis, David Kaufmann, Lisa Umminger, Johanna Seitz-Holland, Nico Sollmann, Yogesh Rathi, Sylvain Bouix, Catherine B. Fortier, David Salat, Ofer Pasternak, Sidney R. Hinds, William P. Milberg, Regina E. McGlinchey, Martha E. Shenton, Inga K. Koerte

**Affiliations:** 1 Psychiatry Neuroimaging Laboratory, Department of Psychiatry, Brigham and Women’s Hospital, Boston, Massachusetts; 2 Department of Neurology, University Hospital, Ludwig-Maximilians-Universität, Munich, Germany; 3 cBRAIN, Department of Child and Adolescent Psychiatry, Psychosomatics, and Psychotherapy, Ludwig-Maximilians-Universität, Munich, Germany; 4 Department of Radiology, Brigham and Women’s Hospital, Harvard Medical School, Boston, Massachusetts; 5 Department of Biostatistics, Boston University School of Public Health, Boston, Massachusetts; 6 Boston University Alzheimer’s Disease and CTE Center, Boston University School of Medicine, Boston, Massachusetts; 7 Department of Diagnostic and Interventional Radiology and Neuroradiology, Klinikum Augsburg, Germany; 8 Department of Diagnostic and Interventional Neuroradiology, School of Medicine, Klinikum rechts der Isar, Technical University of Munich, Munich, Germany; 9 TUM-Neuroimaging Center, Klinikum rechts der Isar, Technical University of Munich, Munich, Germany; 10 Department of Diagnostic and Interventional Radiology, University Hospital Ulm, Ulm, Germany; 11 Translational Research Center for TBI and Stress Disorders and Geriatric Research, Education and Clinical Center, VA Boston Healthcare System, Boston, Massachusetts; 12 Department of Psychiatry, Harvard Medical School, Boston, Massachusetts; 13 Neuroimaging Research for Veterans Center, VA Boston Healthcare System, Boston, Massachusetts; 14 Athinoula A. Martinos Center for Biomedical Imaging, Department of Radiology, Massachusetts General Hospital, Charlestown, Massachusetts; 15 Department of Neurology, Uniformed Services University of the Health Science, Bethesda, Maryland; 16 Department of Psychiatry, Massachusetts General Hospital, Harvard Medical School, Boston, Massachusetts

## Abstract

**Importance:**

Military service members returning from theaters of war are at increased risk for mental illness, but despite high prevalence and substantial individual and societal burden, the underlying pathomechanisms remain largely unknown. Exposure to high levels of emotional stress in theaters of war and mild traumatic brain injury (mTBI) are presumed factors associated with risk for the development of mental disorders.

**Objective:**

To investigate (1) whether war zone–related stress is associated with microstructural alterations in limbic gray matter (GM) independent of mental disorders common in this population, (2) whether associations between war zone–related stress and limbic GM microstructure are modulated by a history of mTBI, and (3) whether alterations in limbic GM microstructure are associated with neuropsychological functioning.

**Design, Setting, and Participants:**

This cohort study was part of the TRACTS (Translational Research Center for TBI and Stress Disorders) study, which took place in 2010 to 2014 at the Veterans Affair Rehabilitation Research and Development TBI National Network Research Center. Participants included male veterans (aged 18-65 years) with available diffusion tensor imaging data enrolled in the TRACTS study. Data analysis was performed between December 2017 to September 2021.

**Exposures:**

The Deployment Risk and Resilience Inventory (DRRI) was used to measure exposure to war zone–related stress. The Boston Assessment of TBI-Lifetime was used to assess history of mTBI. Stroop Inhibition (Stroop-IN) and Inhibition/Switching (Stroop-IS) Total Error Scaled Scores were used to assess executive or attentional control functions.

**Main Outcomes and Measures:**

Diffusion characteristics (fractional anisotropy of tissue [FA_T_]) of 16 limbic and paralimbic GM regions and measures of functional outcome.

**Results:**

Among 384 male veterans recruited, 168 (mean [SD] age, 31.4 [7.4] years) were analyzed. Greater war zone–related stress was associated with lower FA_T_ in the cingulate (DRRI-combat left: *P* = .002, partial *r* = −0.289; DRRI-combat right: *P* = .02, partial *r* = −0.216; DRRI-aftermath left: *P* = .004, partial *r* = −0.281; DRRI-aftermath right: *P* = .02, partial *r* = −0.219), orbitofrontal (DRRI-combat left medial orbitofrontal cortex: *P* = .02, partial *r* = −0.222; DRRI-combat right medial orbitofrontal cortex: *P* = .005, partial *r* = −0.256; DRRI-aftermath left medial orbitofrontal cortex: *P* = .02, partial *r* = −0.214; DRRI-aftermath right medial orbitofrontal cortex: *P* = .005, partial *r* = −0.260; DRRI-aftermath right lateral orbitofrontal cortex: *P* = .03, partial *r* = −0.196), and parahippocampal (DRRI-aftermath right: *P* = .03, partial *r* = −0.191) gyrus, as well as with higher FA_T_ in the amygdala-hippocampus complex (DRRI-combat: *P* = .005, partial *r* = 0.254; DRRI-aftermath: *P* = .02, partial *r* = 0.223). Lower FA_T_ in the cingulate-orbitofrontal gyri was associated with impaired response inhibition (Stroop-IS left cingulate: *P* < .001, partial *r* = −0.440; Stroop-IS right cingulate: *P* < .001, partial *r* = −0.372; Stroop-IS left medial orbitofrontal cortex: *P* < .001, partial *r* = −0.304; Stroop-IS right medial orbitofrontal cortex: *P* < .001, partial *r* = −0.340; Stroop-IN left cingulate: *P* < .001, partial *r* = −0.421; Stroop-IN right cingulate: *P* < .001, partial *r* = −0.300; Stroop-IN left medial orbitofrontal cortex: *P* = .01, partial *r* = −0.223; Stroop-IN right medial orbitofrontal cortex: *P* < .001, partial *r* = −0.343), whereas higher FA_T_ in the mesial temporal regions was associated with improved short-term memory and processing speed (left amygdala-hippocampus complex: *P* < .001, partial *r* = −0.574; right amygdala-hippocampus complex: *P* < .001, partial *r* = 0.645; short-term memory left amygdala-hippocampus complex: *P* < .001, partial *r* = 0.570; short-term memory right amygdala-hippocampus complex: *P* < .001, partial *r* = 0.633). A history of mTBI did not modulate the association between war zone–related stress and GM diffusion.

**Conclusions and Relevance:**

This study revealed an association between war zone–related stress and alteration of limbic GM microstructure, which was associated with cognitive functioning. These results suggest that altered limbic GM microstructure may underlie the deleterious outcomes of war zone–related stress on brain health. Military service members may benefit from early therapeutic interventions after deployment to a war zone.

## Key Points

**Question:**

Is war zone–related stress associated with limbic gray matter (GM) microstructure?

**Findings:**

In this cohort study of US veterans, exposure to war zone–related stress was associated with alterations in limbic GM microstructure, independent of the diagnosis of mental disorder or mild traumatic brain injury. Furthermore, GM microstructure was associated with cognitive functioning.

**Meaning:**

These findings suggest that war zone–related stress may lead to limbic GM microstructure alterations, which may underlie the deleterious outcomes of war zone–related stress on brain health and that military service members may benefit from early therapeutic interventions following deployment.

## Teaser

This cohort study evaluates the association between war zone–related stress and alterations in limbic gray matter microstructure in a group of veterans from the US.

## Introduction

Military personnel serving in theaters of war are at increased risk for physical and mental health problems following deployment.^1,2,3^ Mental health–related disorders are pervasive; up to 30% of service members returning from Operation Enduring Freedom (OEF), Operation Iraqi Freedom (OIF), or Operation New Dawn (OND) receive a diagnosis of a mental illness, such as posttraumatic stress disorder (PTSD), anxiety, or depression.^4,5,6^ Known factors associated with postdeployment mental disorders include combat exposure and associated psychosocial stressors.^7,8,9^ Importantly, service members exhibit symptoms related to war zone stress and experience low quality of life even if they do not meet the diagnostic criteria for a mental disorder.^10^ Furthermore, despite the prevalence and adversity of war zone–related stress, the majority of previous studies have not specifically investigated the impact of war zone–related stress, and even fewer have used quantitative questionnaires such as the Deployment Risk and Resilience Inventory (DRRI) to quantify perceived war zone–related stress.^11,12,13,14^ Although mental health problems are highly prevalent in postdeployed military service members^15^ and war zone–related stress has been discussed as a risk factor, the underlying pathomechanisms remain poorly understood.

Furthermore, approximately 12% to 35% of OEF, OIF, and OND veterans have sustained a mild traumatic brain injury (mTBI).^16,17,18,19^ Evidence suggests that mTBI is not only a highly prevalent comorbidity but is also considered a potential risk factor for the development of mental disorders. In fact, service members who have sustained mTBI have a significantly increased risk for developing PTSD^1,16,20,21,22^ and depression.^1,23,24^ Moreover, they exhibit poorer neurocognitive functioning, worse long-term recovery,^25^ and more severe neurological impairment^26,27^ compared with those who have not sustained mTBI. However, it is unknown whether comorbidity with mTBI modulates a possible association between war zone–related stress and alterations of brain structure and neuropsychological functioning. A better understanding of the outcomes of war zone–related stress on brain microstructure and function is critical for improving long-term health and quality of life of military service members returning from theaters of war.

Magnetic resonance imaging (MRI) provides a noninvasive way to study brain alterations as it allows for the in vivo, 3-dimensional investigation of brain macrostructure and microstructure.^28^ Neuroimaging studies have linked neuropsychiatric disorders, including PTSD and mTBI, to macrostructural brain alterations.^29^ However, although an association between diagnoses and abnormal brain structure has been established, research on the outcomes of war zone–related stress on brain structure is sparse. Combat exposure has been found to be associated with lower volume of limbic or limbic-associated gray matter (GM) regions, such as the amygdala,^30^ hippocampus,^31,32^ orbitofrontal gyrus,^33^ posterior insula,^34^ ventromedial prefrontal cortex, and dorsal anterior cingulate cortex.^35^ Of note, although lower limbic GM volumes have been associated with PTSD symptom severity and extent of alcohol use, other disorders commonly seen in this population have previously not been considered.

Diffusion-weighted MRI (dMRI) has been shown to be sensitive to subtle microstructural brain alterations associated with neuropsychiatric disorders, such as PTSD and mTBI.^29^ Complementary to volumetric measures, dMRI has the potential to reveal alterations in tissue composition (eg, glial changes^36,37,38^ and atrophy^39^) and tissue morphologic changes (eg, alterations in dendritic arborization^40,41,42^), thereby providing insight into underlying pathomechanisms. Although most research to date has focused on the microstructure of connecting white matter (WM) fiber tracts,^43,44,45,46^ studies on the limbic GM microstructure are sparse. Importantly, to our knowledge, no study to date has investigated the association between combat exposure and limbic GM diffusion, although limbic GM constitutes an essential neuroanatomical correlate of mental and neuropsychological functioning as suggested previously by volumetric studies^31,32,47^ of limbic system structures in postdeployed veterans. The aim of this study is to investigate (1) whether war zone–related stress is associated with microstructural alterations in limbic GM independent of mental disorders, (2) whether associations between war zone–related stress and limbic GM microstructure are modulated by a history of mTBI, and (3) whether alterations in limbic system GM microstructure are associated with neuropsychological functioning.

## Methods

This cohort study was approved by the institutional review board of human studies research at the Veterans Affair Boston Healthcare System and all participants provided written informed consent. The study follows the Strengthening the Reporting of Observational Studies in Epidemiology (STROBE) reporting guideline for observational studies.

### Participants

The Translational Research Center for TBI and Stress Disorders (TRACTS) study is a longitudinal prospective cohort study that aims to assess and track the potential outcomes of psychologically and physically traumatic experiences related to military deployment over time. Inclusion criteria for enrollment into the TRACTS study were (1) age 18 to 65 years, (2) male sex, and (3) service in OEF, OIF, or OND, or scheduled deployment.^48^ Exclusion criteria were (1) history of neurological illness other than TBI; (2) current diagnosis of schizophrenia spectrum or other psychotic disorders; (3) current diagnosis of bipolar or related disorders; (4) active suicidal and/or homicidal ideation, intent, or plan requiring crisis intervention; and (5) cognitive disorder due to general medical condition other than TBI. Parameters with potential impact on cerebral microstructure and resilience such as eduction, socioeconomic status, race and ethnicity were collected via interview.

**Figure 1.**
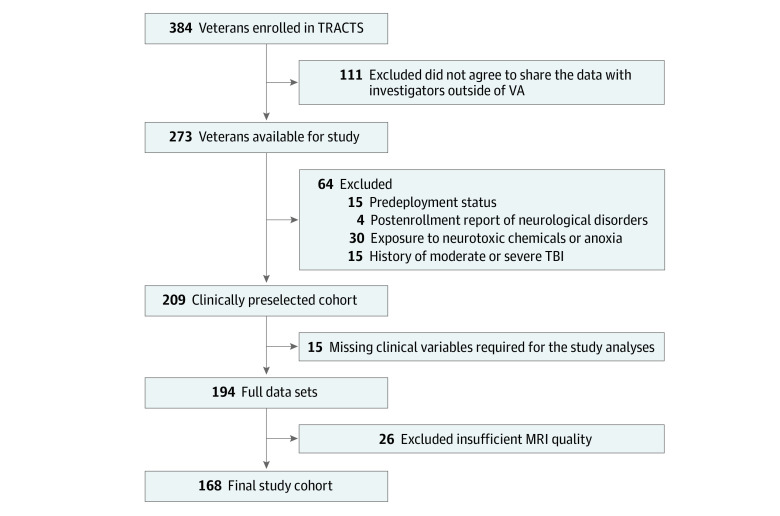
Flowchart of the Cohort Selection Process MRI indicates magnetic resonance imaging; TBI traumatic brain injury; TRACTS, Translational Research Center for TBI and Stress Disorders; VA, Veterans Affairs.

Of the first 384 consecutively recruited veterans, 273 consented to share their data with investigators outside of TRACTS. Of these 273 veterans, several had to be excluded from the present study for the following reasons: predeployment status (ie, military service members who had not yet been deployed to combat zones) (15 participants), postenrollment report of neurological disorders (ie, history of meningitis, or brain surgery; 4 participants), history of moderate or severe TBI (15 participants), and exposure to neurotoxic chemicals or anoxia (30 participants). Another 26 cases did not pass the rigorous quality control of the MRI data, and 15 cases had missing clinical variables required for this study. The selection process is summarized in Figure 1.

### Diagnostic and Clinical Assessment

#### Assessment of Psychiatric Disorders

The nonpatient edition of the Structured Clinical Interview for DSM-IV Axis I Disorders (SCID-I/NP)^49^ was used to detect the presence of psychopathological disorders. The following modules were administered: module D, mood disorders; module E, substance use disorders; module F, anxiety disorders (except PTSD); module H, eating disorders; and module I, adjustment disorders. Presence and history of PTSD were determined according to the Clinician-Administered PTSD Scale (CAPS)^50^ using the *Diagnostic and Statistical Manual of Mental Disorders (Fourth Edition)* (*DSM-IV*) standard scoring rule.^51^

#### Assessment of mTBI

The Boston Assessment of TBI-Lifetime (BAT-L)^17^ was conducted to diagnose lifetime history of TBI. Specifically, mTBI was defined by the following criteria: loss of consciousness for 30 minutes or less, posttraumatic amnesia for 24 hours or less, or altered mental status for 24 hours or less.^17^

#### Assessment of War Zone–Related Stress

Stressors associated with deployment to war zones were assessed via selected scales from the DRRI.^52^ The combat experiences and aftermath of battle scales were used to assess perceived war zone–related stress. Both DRRI subscales (called hereafter DRRI-combat and DRRI-aftermath) consist of 16 questions concerning combat or war zone–related events. The DRRI-combat uses a 5-point Likert frequency scale (0 = never; 4 = daily or almost daily), yielding a maximum possible score of 64 points. The DRRI-aftermath scale uses a binary response (0 = no and 1 = yes), resulting in a maximum score of 16 points. Higher scores on both the DRRI-combat and DRRI-aftermath scale reflect greater exposure to deployment-related stressors.

#### Assessment of Functional Outcome

The World Health Organization Disability Assessment Schedule II (WHODAS II)^53^ is a 36-item self-report questionnaire that was designed to measure disability associated with all physical and mental disorders including cognition, mobility, self-care, getting along, life activities, and participation. Functional impairments within the last 30 days are rated on a 5-point scale (0 = no disability; 4 = extreme disability/cannot do). A total disability score is calculated by summing the scores across all subscales. Higher scores reflect greater disability.

The Neurobehavioral Symptom Inventory (NSI) is a 22-item self-report questionnaire used to assess postconcussion symptoms following TBI.^54^ Tested symptoms include sensory, affective, vestibular, and cognitive symptoms, rated on a 5-point Likert scale (0 = none; 4 = very severe). Higher scores reflect more severe neurobehavioral symptoms.

According to identified limbic regions with GM diffusion alterations, the Digit Span Total Score (DSTot) and the Coding Raw Scores^55^ were chosen from the comprehensive neuropsychological test battery,^48^ as they reflect functions of the frontal and temporal lobe (ie, verbal short-term memory performance and processing speed). In addition, Stroop Inhibition (Stroop-IN) and Inhibition/Switching (Stroop-IS) Total Error Scaled Scores^56^ were selected to assess more specifically executive or attentional control functions associated with the prefrontal and cingulate cortex,^57,58,59,60,61,62^ whereby higher Total Error Scaled Scores reflect impaired response inhibition and vice versa.

#### Assessment of Hypervigilance

The CAPS criterion D was used to assess the frequency and intensity of symptoms of hypervigilance at postdeployment, including difficulty sleeping, irritability, difficulty concentrating, hypervigilance, and exaggerated startle response. Answers were rated on a 5-point Likert scales ranging from 0 to 4 and summarized in a total score, resulting in a maximum score of 40 points.

#### Effort Testing

Performance validity was assessed via the Verbal Multiple Symptom Validity Test (MSVT).^63^ The MSVT evaluates verbal learning, memory, and response consistency. It is composed of the subtests immediate recall, delayed recognition, consistency of responding across immediate recall, and delayed recognition, as well as paired associates and free recall. Study participants who failed the MSVT (8 participants) were excluded from the post hoc analyses as they were suspected of potential reduced effort or malingering.

#### MRI Acquisition and Data Processing

MRI of the brain was performed using a 3-Tesla TIM Trio scanner (Siemens Healthineers) located at the VA Medical Center in Boston, Massachusetts. T1-weighted (T1w) gradient-echo sequence parameters were field of view, 256 mm; 256 sections; inversion time, 1.000 ms; repetition time, 2.530 ms; echo time, 3.32 ms; flip angle, 7°; and isotropic resolution, 1 × 1 × 1 mm^3^. dMRI was acquired using a single-shot, echo-planar sequence with a twice-refocused spin-echo pulse and the following parameters: field of view, 256 mm; 64 axial sections with no intersection gap; 60 gradient directions with a b-value of 700 seconds/mm^2^; 10 b = 0 volumes; repetition time, 10 000 ms; echo time, 103 ms; and isotropic resolution, 2 × 2 × 2 mm^3^.

dMRI data were corrected for motion and eddy current distortions via affine registration to the first b = 0 volume using FMRIB Software Library, version 5.1 (The Oxford Centre for Functional MRI of the Brain).^64^ Brain masks were created and manually edited in 3D Slicer, version 4.5 (Surgical Planning Laboratory, Brigham and Women´s Hospital).^65^ Automated segmentation of brain regions from the T1w data was performed using FreeSurfer^66^ (version 5.1.0).^67^

Free water (FW)–corrected diffusion tensor measures were derived from dMRI using in-house software.^68^ FW imaging separates the dMRI signal into 2 compartments: a FW and a tissue compartment. FW in the brain is expected where water molecules are free to diffuse, such as in cerebrospinal fluid, and large extracellular spaces. We calculated a fractional anisotropy of tissue (FA_T_) map from the FW-corrected diffusion tensor, which serves as a more accurate marker of anisotropy in brain tissue than the conventional FA measure. To obtain diffusion metrics for selected regions, FreeSurfer parcellation label maps were nonlinearly registered from the individual T1w space to the respective dMRI space to obtain diffusion metrics for selected regions. Eight limbic and paralimbic GM regions in each hemisphere were evaluated—that is, cingulate gyrus, amygdala-hippocampus complex, parahippocampal gyrus, entorhinal cortex, lateral and medial orbitofrontal cortex, insula, and temporal pole. Amygdala and hippocampus were combined into 1 region of interest to ensure higher parcellation accuracy.^69^ For each of these 8 bihemispheric regions of interest (16 in total), the mean of the diffusion measure (FA_T_) was calculated.

### Statistical Analysis

Statistical analyses were performed using SAS statistical software version 9.4 (SAS Institute). Means and SDs are displayed for continuous parameters, while absolute and relative frequencies are provided for noncontinuous variables. Generalized linear models for repeated measures using the restricted maximum likelihood approach and an unstructured covariance matrix across brain regions were used to evaluate the association of war zone–related stress with regional diffusion measures. The following parameters were selected a priori as covariates: age, diagnosis of current PTSD, mood, anxiety, substance use disorder, and weight-corrected lifetime drinking history (LDH). To test the outcomes of mTBI on the association between war zone–related stress and limbic GM diffusion, the number of lifetime mTBIs was added as fixed effect as well as modifier to the main effect.

Post hoc analyses were conducted to test for associations between diffusion measures that were significantly associated with war zone stress and neurobehavioral symptoms (NSI), cognitive (DSTot, Coding Raw Score, and Stroop IN/IS Total error scaled score), and disability (WHODAS). Participants who failed error testing (MSVT) were excluded from the post hoc analyses. Age, diagnosis of current PTSD, mood, anxiety, and substance use disorder, and LDH were included as covariates.

A false discovery rate^70^ was set at 5% to correct for multiple comparisons, using the Benjamini-Hochberg method. A corrected 2-tailed *P* < .05 was considered significant. Data were analyzed December 2017 to September 2021.

## Results

The final study cohort encompassed 168 male veterans with a mean (SD) age of 31.4 (7.4) years. Sample demographic characteristics are summarized in Table 1. The vast majority of participants were White (130 participants [77%]), followed by 24 Hispanic participants (14%) and 11 Black participants (6%) (Table 1). Although the level of education was balanced across the cohort (mean [SD] 13.9 [1.9] school years), potentially relevant differences were observed for the family status as only 38% (64 participants) were married or cohabiting.

**Table 1.** Demographics, Deployment-Related Factors, and Postdeployment Characteristics of Study Cohort

Variable	Participants, No. (%) (N = 168)
Demographics	
Age, mean (SD), y	31.36 (7.43)
Race and ethnicity	
Asian	2 (1.19)
Black	11 (6.55)
Hispanic	24 (14.29)
Unknown	1 (0.60)
White	130 (77.38)
Education mean (SD), school years	13.86 (1.93)
Married or cohabitating	64 (38.10)
Deployment factors	
OEF, OIF, or OND deployments, mean (SD), No.	1.4 (0.7)
Other stressful deployments, mean (SD), No.	0.41 (0.79)
Duration of OEF, OIF, or OND deployments, mean (SD), mo	13.82 (8.45)
Service in army branch	101 (60.12)
DRRI total score, mean (SD)	
Combat experience (DRRI-combat)	17.31 (12.02)
Aftermath exposure (DRRI-aftermath)	7.65 (4.7)
Military mTBIs, mean (SD), No.	0.63 (1.53)
Wounded or injured in combat	35 (20.83)
Postdeployment characteristics	
Time since last deployment, mean (SD), mo	40.07 (29.98)
Disorder	
Mood	35 (20.83)
Anxiety	28 (16.67)
PTSD diagnosis	112 (66.67)
Clinician-Administered PTSD Scale, mean (SD)^a^	78.35 (22.9)
Substance use disorder	25 (14.88)
Lifetime drinking history, weight corrected, mean (SD)	1790.6 (2092.7)
Lifetime TBIs, mean (SD)	1.38 (2.23)

Abbreviations: DRRI, Deployment Risk and Resilience Inventory; mTBI, mild traumatic brain injury; OEF, Operation Enduring Freedom; OIF, Operation Iraqi Freedom; OND, Operation New Dawn; PTSD, posttraumatic stress disorder; TBI, traumatic brain injury.

^a^ Clinician-Administered PTSD Scale score was evaluated for 112 veterans who met diagnostic criteria for postdeployment PTSD.

### Associations of War Zone–Related Stress With Limbic GM Diffusion

In the cohort of 168 veterans, greater war zone–related stress as assessed by DRRI-combat and DRRI-aftermath was negatively associated with FA_T_ in the bilateral cingulate gyri (DRRI-combat left: *P* = .002, partial *r* = −0.289, *df* = 167; DRRI-combat right: *P* = .02, partial *r* = −0.216, *df* = 167; DRRI-aftermath left: *P* = .004, partial *r* = −0.281, *df* = 167; DRRI-aftermath right: *P* = .02, partial *r* = −0.219, *df* = 167) and bilateral medial orbitofrontal gyri (DRRI-combat left medial orbitofrontal cortex: *P* = .02, partial *r* = −0.222, *df* = 167; DRRI-combat right medial orbitofrontal cortex: *P* = .005, partial *r* = −0.256, *df* = 167; DRRI-aftermath left medial orbitofrontal cortex: *P* = .02, partial *r* = −0.214, *df* = 167; DRRI-aftermath right medial orbitofrontal cortex: *P* = .005, partial *r* = −0.260, *df* = 167; DRRI-aftermath right lateral orbitofrontal cortex: *P* = .03, partial *r* = −0.196, *df* = 167). Notably, these associations were observed while controlling for age, PTSD diagnosis, mood disorder, anxiety disorder, and substance use disorder as well as LDH.

Moreover, a negative association was observed between DRRI-aftermath and the right lateral orbitofrontal gyrus FA_T_ and right parahippocampal gyrus FA_T_ (*P* = .03, partial *r* = −0.191, *df* = 167). In contrast, a positive association was found for both measures of war zone–related stress and FA_T_ in the right amygdala-hippocampus complex (DRRI-combat: *P *= .005, partial *r* = 0.254, *df* = 167; DRRI-aftermath: *P* = .02, partial *r* = 0.223, *df* = 167). Results are summarized in Table 2.

**Table 2.** Association of War Zone–Related Stress and Limbic Gray Matter Diffusion Using Fractional Anisotropy of Tissue

Region	Combat exposure (DRRI-combat)				Aftermath exposure (DRRI-aftermath)			
	Left hemisphere		Right hemisphere		Left hemisphere		Right hemisphere	
	Partial *r*^a^	FDR corrected *P* value	Partial *r*^a^	FDR corrected *P* value	Partial *r*^a^	FDR corrected *P* value	Partial *r*^a^	FDR corrected *P* value
Amygdala-hippocampus complex	0.158	.09	0.254	.005^b^	0.136	.14	0.224	.02^b^
Cingulate gyrus	−0.289	.002^b^	−0.216	.02^b^	−0.281	.004^b^	−0.219	.02^b^
Entorhinal cortex	0.020	.80	0.121	.21	−0.023	.88	0.049	.65
Insular cortex	−0.058	.52	−0.057	.52	−0.138	.14	−0.061	.57
Lateral orbitofrontal cortex	−0.081	.43	−0.151	.10	−0.083	.41	−0.196	.03^b^
Medial orbitofrontal cortex	−0.222	.02^b^	−0.256	.005^b^	−0.214	.02^b^	−0.260	.005^b^
Parahippocampal gyrus	−0.059	.52	−0.166	.08	−0.009	.97	−0.191	.03^b^
Temporal pole	−0.089	.40	0.053	.52	−0.224	.39	−0.003	.97

Abbreviations: DRRI, Deployment Risk and Resilience Inventory; FDR, false discovery rate.

^a^ The higher the partial *r*, the stronger the linear association between 2 variables. Positive values represent positive correlations, and negative values represent negative or inverse correlations.

^b^ Denotes significant results.

### Outcomes of mTBI on the Association of War Zone–Related Stress and Limbic GM Diffusion

The majority of veterans (109 of 168 [64.9%]) sustained at least 1 mTBI before or during deployment. They reported having experienced a mean (SD) of 1.38 (2.23) mTBIs throughout life with a maximum number of 18 mTBIs. Number of lifetime mTBIs was not associated with limbic GM diffusion and did not mediate the association between war zone–related stress and limbic GM FA_T_.

### Association of Limbic GM Diffusion and Functional Outcome

**Figure 2.**
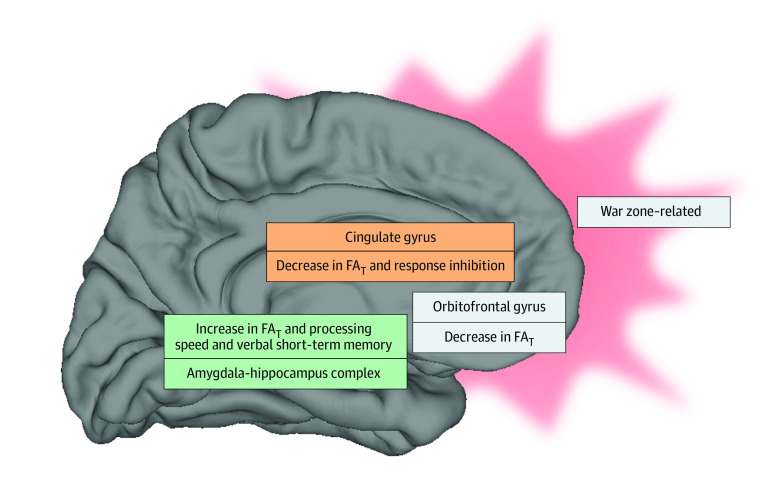
Model of Structural Brain Alterations and Associated Cognitive Function in the Context of War Zone–Related Stress War zone–related stress was associated with decreased fractional anisotropy of tissue (FA_T_) in cingulate and orbitofrontal cortex, as well as increased FA_T_ in the amygdala-hippocampus complex. Limbic gray matter FA_T_ measures were further associated with cognitive function (ie, impaired response inhibition as well as improved verbal short-term memory and processing speed). Taken together with the current literature on functional imaging in posttraumatic stress disorder, we propose that the observed diffusion alterations may result from a functional shift from frontolimbic toward mesial temporal structures (shift of functional demand from cingulate and orbitofrontal regions toward mesial temporal regions).

Results of the post hoc analysis of diffusion and associated functioning are shown in Table 3. Decreased FA_T_ in the cingulate gyri and the medial orbitofrontal cortex was associated with impaired response inhibition (Stroop-IS left cingulate: *P* < .001, partial *r* = −0.440, *df* = 151; Stroop-IS right cingulate: *P* < .001, partial *r* = −0.372, *df* = 151; Stroop-IS left medial orbitofrontal cortex: *P* < .001, partial *r* = −0.304, *df* = 151; Stroop-IS right medial orbitofrontal cortex: *P* < .001, partial *r* = −0.340, *df* = 151; Stroop-IN left cingulate: *P* < .001, partial *r* = −0.421, *df* = 151; Stroop-IN right cingulate: *P* < .001, partial *r* = −0.300, *df* = 151; Stroop-IN left medial orbitofrontal cortex: *P* = .01, partial *r* = −0.223; *df* = 151; Stroop-IN right medial orbitofrontal cortex: *P* < .001, partial *r* = −0.343, *df* = 151), but with better frontotemporal functions (DSTot left amygdala-hippocampus complex: *P* < .001, partial *r* = −0.574, *df* = 159; DSTot right amygdala-hippocampus complex: *P* < .001, partial *r* = 0.645, *df* = 159; short-term memory left amygdala-hippocampus complex: *P* < .001, partial *r* = 0.570, *df* = 156; short-term memory right amygdala-hippocampus complex: *P* < .001, partial *r* = 0.633, *df* = 156). In contrast, impaired response inhibition and improved verbal short-term memory performance and processing speed were associated with increased FA_T_ in the amygdala-hippocampal region (Figure 2). No significant associations were revealed for limbic GM diffusion and (postconcussion) neurobehavioral symptoms or disability (eTable 1 in the Supplement).

**Table 3.** Association of Limbic Gray Matter Diffusion Using Fractional Anisotropy of Tissue and Cognitive Functioning

Region	Digit Span Total Score		Coding Raw Score		Stroop inhibition			
					Total error scaled score		Switching total error scaled score	
	Partial *r*^a^	FDR corrected *P* value	Partial *r*^a^	FDR corrected *P* value	Partial *r*^a^	FDR corrected *P* value	Partial *r*^a^	FDR corrected *P* value
Left amygdala-hippocampus comp	0.574	<.001^b^	0.570	<.001^b^	0.443	<.001^b^	0.483	<.001^b^
Left cingulate gyrus	−0.393	<.001^b^	−0.330	<.001^b^	−0.421	<.001^b^	−0.440	<.001^b^
Left lateral orbitofrontal cortex	−0.058	.74	−0.006	.94	−0.036	.79	−0.044	.78
Left medial orbitofrontal cortex	−0.202	.02^b^	−0.193	.03^b^	−0.223	.01^b^	−0.304	<.001^b^
Left parahippocampal gyrus	0.042	.80	0.007	.94	0.059	.79	0.013	.95
Right amygdala-hippocampus comp	0.645	<.001^b^	0.633	<.001^b^	0.500	<.001^b^	0.518	<.001^b^
Right cingulate gyrus	−0.290	<.001^b^	−0.237	.007^b^	−0.300	<.001^b^	−0.372	<.001^b^
Right lateral orbitofrontal cortex	0.041	.80	0.024	.76	−0.038	.79	0.005	.95
Right medial orbitofrontal cortex	−0.263	.002^b^	−0.262	.003^b^	−0.343	<.001^b^	−0.340	<.001^b^
Right parahippocampal gyrus	−0.001	.99	−0.021	.79	−0.032	.79	−0.103	.35

Abbreviation: FDR false discovery rate.

^a^ The higher the partial *r*, the stronger the linear association between 2 variables. Positive values represent positive, and negative values represent negative or inverse correlations.

^b^ Denotes significant results.

### Association of Limbic GM Diffusion and Hypervigilance State

Hypervigilance at postdeployment was positively associated with FA_T_ in the amygdala-hippocampal region (left: *P* < .001, partial *r* = 0.325, *df* = 165; right: *P* <.001, partial *r* = 0.309; *df* = 165) and negatively associated with FA_T_ in the cingulate gyri (left: *P* < .01, partial *r* = −0.253 *df* = 165; right: *P* < .01; partial *r* = −0.261 *df* = 165). The results are summarized in eTable 2 in the Supplement.

## Discussion

This cohort study found an association between war zone–related stress and microstructure of limbic GM in veterans. Importantly, these findings were observed while accounting for common comorbidities, including PTSD, mood, anxiety, and substance use disorder. Furthermore, mTBI had no significant effect on the association between war zone–related stress and limbic GM microstructure. Finally, characteristics of limbic GM microstructure were associated with cognitive performance including verbal short-term memory, processing speed, and response inhibition, while no associations with overall disability and neurobehavioral symptoms were found.

### War Zone–Related Stress and Limbic GM Diffusion

This study revealed a co-occurring decrease and increase in limbic GM FA_T_. More specifically, the greater the experienced war zone–related stress, the lower FA_T_ was in the cingulate gyri, the medial orbitofrontal gyri, the right lateral orbitofrontal gyrus, and the right parahippocampal gyrus. Moreover, the greater the experienced war zone–related stress, the higher the FA_T_ in the amygdala-hippocampus complex. Importantly, associations described previously were independent of diagnosis of mental disorders as well as mTBI.

The interpretation of diffusion measures in GM is challenging as data linking diffusion to histologic profile is sparse.^37,71,72,73,74^ FA in GM likely reflects diffusion properties of the main GM components (ie, astroglia, neurons, and axons). For example, a study in mice reveals an association between decreased FA and decreased astrocyte density in the hippocampus.^71^ Astrocytes play a crucial role in complex brain functions, such as neurotransmitter homeostasis and blood-brain barrier maintenance.^75^ Moreover, a decrease in astrocytes predisposes the brain to inflammatory states.^75,76^ Another dMRI study^77^ in a murine model of Parkinson disease found an association between decreased FA_T_ in the substantia nigra and neuronal loss. Taken together, the association between war zone–related stress and decreased FA_T_ in the cingulate, orbitofrontal gyri, and right parahippocampal gyrus may potentially be due to a decrease in astrocytes and/or neurons.

Interestingly, a positive association was found for greater war zone–related stress and higher FA_T_ in the amygdala-hippocampus complex. Increased FA_T_ in GM and WM has been associated with neuroplastic remodeling.^72,73^ In rodents, long-term learning and memory tasks induced an FA increase, particularly in limbic system structures such as the amygdala, the parahippocampal gyrus, and the cingulate cortex, which correlated with an increase in a myelin marker (myelin basic protein) in the histological analysis.^72,73^ The authors^72,73^ hypothesized that oligodendrocytes, which form the myelin sheaths in the central nervous system, produced more myelin basic protein postlearning to allow for the required flow of information. Taken together, findings of our study suggest regional differences in the association between war zone–related stress and alterations in GM microstructure that may be due to neurodegenerative and neuroplastic processes.

### Association Between Limbic GM Diffusion and Functional Outcome

We observed improved frontotemporal brain functions (ie, short-term memory and processing speed) in association with increased FA_T_ in the amygdala-hippocampal complex (Figure 2), which is in line with previous studies that report a link between processing speed and hippocampal FA.^78,79,80^ Our study results further suggest an association between improved frontotemporal brain functions with war zone–related stress.^81^ It has been hypothesized that hypervigilance and readiness to respond to combat-related challenges may be advantageous adaptations to the highly stressful environment. However, it may be challenging to transition back to normal states of alertness when returning from deployment. The chronic activated state may consequently lead to a functional overuse of frontotemporal brain functions. This overuse may induce neuroplastic changes as suggested by the increased FA_T_ in the amygdala-hippocampal complex^73^ found in this study. This hypothesis is supported by our finding of a significant association between hypervigilance state at postdeployment and increased FA_T_ in the amygdala-hippocampal complex.

At the same time, we observed impaired prefrontal-cingulate functions (response inhibition) in association with lower FA_T_ in prefrontal regions. This is thought to result from functional (emotional or stress) overuse of mesial temporal structures, as described previously, which may, in turn, lead to poorer performance in other cognitive tasks, a phenomenon called interference.^82,83,84^ Interference or shift of emotion and cognition has previously been described in patients with PTSD^85^ as well as in veterans. More specifically, impaired memory consolidation and reduced learning speed were observed in veterans returning from OEF, OIF, or OND.^86,87^ Of note, those functions are typically associated with the prefrontal-cingulate cortex,^86,87,88,89^ regions that have been found to have lower FA_T_ in association with war zone stress in the current study.

Taken together, we hypothesize that the outcomes of war zone–related stress outlast deployment, leading to attentional interference with increased functional use of mesial temporal structures and decreased use or impaired retrieval of prefrontal-cingulate functions. This hypothesis is further supported by functional MRI studies,^90^ which have reported a hypoconnectivity of mesial temporal and prefrontal brain regions under conditions of stress. The functional interference may, in turn, lead to microstructural adaptations, reflected by increased FA_T_ in the amygdala-hippocampus complex and decreased FA_T_ in the cingulate and orbitofrontal gyri (Figure 2). This biological adaptive response may potentially, in addition to preexisting biological predisposition for deployment, mean that service members with outstanding processing speed and verbal short-term memory might be more likely to join the military and to be deployed.

No significant associations were found between limbic GM diffusion and more general measures of functional outcome following mTBI (ie, the WHODAS and NSI). We thus speculate that abnormalities in the limbic system may need to be more severe to cause impairments in everyday functioning. Furthermore, the observed limbic alterations may represent a minor contributor to everyday functioning as assessed using WHODAS and NSI, whereas the individual comorbidities may be the main drivers of the functional impairment.

### Limitations

Our study has limitations. We investigated a representative subsample of OEF, OIF, or OND veterans^48^ and we accounted for common comorbidities in the statistical analysis. However, we used dichotomous variables based on the *DSM-IV* classifications to account for the presence of psychopathologic disorders. Future studies should consider using dimensional assessments of psychopathologic disorders, to further investigate the spectrum of psychopathologic disorders. Furthermore, we did not account for service branch, race, or socioeconomic status,^91,92,93,94,95,96,97,98^ which might be of importance for resilience, stress exposure, management, and rehabilitation and should be considered in future analyses. The vast majority of participants were White, followed by Hispanic and Black participants (Table 1). Although the level of education was balanced across the cohort, potentially relevant differences were observed for the family status as only 38% were married or cohabiting. A further limitation is that this study was limited to male participants only. The cross-sectional design of this study further limits the interpretation of our findings as well as the identification of additional factors associated with risk and causal relationship between war zone–related stress and alterations in limbic GM may not be drawn. Moreover, we did not differentiate between the amygdala and hippocampus as we aimed for the highest possible accuracy in the segmentation. Previous research of imaging data has demonstrated that the use of the combined amygdala-hippocampus complex represents a methodologically more rigorous and accurate approach of segmentation using FreeSurfer.^69^ Against the background of our study findings, future studies should strive to retest our hypothesis on manually segmented limbic GM. Additionally, although all interviews were conducted by doctoral level psychologists, their administration at long-term follow-up might have been inevitably biased by participant subjective memory and reporting. Of further note, multishell dMRI data would have improved the FW model fit but was not available in the study. In addition, the analysis of GM is highly sensitive to misalignment of the diffusion space and T1 space, which may have caused inflation in the FW measure. Despite the FW-correction, the FA_T_ measures remain unspecific and can only serve as a gross estimation of the underlying microstructure.

## Conclusions

In this study, war zone–related stress was associated with alterations in limbic GM microstructure, which, in turn, were associated with cognitive function independent of the diagnosis of mental disorders and mTBI commonly observed in this population. Taken together, findings from this study suggest that alterations in limbic GM microstructure may underlie the deleterious outcomes of exposure to war zone–related stress. Thus, military service members exposed to war zone–related stress may benefit from early therapeutic intervention even in the absence of a diagnosed mental disorder.

## Notes

Supplement. **eTable 1.** Association of Limbic Gray Matter Diffusion (FAT) and Disability/Neurobehavioral Symptoms **eTable 2.** Association of Limbic Gray Matter Diffusion (FAT) and Hypervigilance State

## References

[1] Lippa SM, Fonda JR, Fortier CB, . Deployment-related psychiatric and behavioral conditions and their association with functional disability in OEF/OIF/OND veterans. J Trauma Stress. 2015;28(1):25-33. doi:10.1002/jts.21979 25703936 PMC5556936

[2] Elder GH Jr, Shanahan MJ, Clipp EC. Linking combat and physical health: the legacy of World War II in men’s lives. Am J Psychiatry. 1997;154(3):330-336. doi:10.1176/ajp.154.3.330 9054779

[3] Wolfe J, Schnurr PP, Brown PJ, Furey J. Posttraumatic stress disorder and war-zone exposure as correlates of perceived health in female Vietnam War veterans. J Consult Clin Psychol. 1994;62(6):1235-1240. doi:10.1037/0022-006X.62.6.1235 7860822

[4] Hoge CW, Castro CA, Messer SC, McGurk D, Cotting DI, Koffman RL. Combat duty in Iraq and Afghanistan, mental health problems, and barriers to care. N Engl J Med. 2004;351(1):13-22. doi:10.1056/NEJMoa040603 15229303

[5] Tanielian TL, Jaycox LH, eds. Invisible Wounds of War: Psychological and Cognitive Injuries, Their Consequences, and Services to Assist Recovery. RAND Corporation; 2008.

[6] Ramchand R, Rudavsky R, Grant S, Tanielian T, Jaycox L. Prevalence of, risk factors for, and consequences of posttraumatic stress disorder and other mental health problems in military populations deployed to Iraq and Afghanistan. Curr Psychiatry Rep. 2015;17(5):37. doi:10.1007/s11920-015-0575-z 25876141

[7] Larson GE, Booth-Kewley S, Highfill-McRoy RM, Young SYN. Prospective analysis of psychiatric risk factors in marines sent to war. Mil Med. 2009;174(7):737-744. doi:10.7205/MILMED-D-02-0308 19685846

[8] Booth-Kewley S, Schmied EA, Highfill-McRoy RM, Larson GE, Garland CF, Ziajko LA. Predictors of psychiatric disorders in combat veterans. BMC Psychiatry. 2013;13(1):130. doi:10.1186/1471-244X-13-130 23651663 PMC3651311

[9] Dohrenwend BP, Turner JB, Turse NA, Adams BG, Koenen KC, Marshall R. The psychological risks of Vietnam for U.S. veterans: a revisit with new data and methods. Science. 2006;313(5789):979-982. doi:10.1126/science.1128944 16917066 PMC1584215

[10] Brooks DE, Agochukwu UF, Arrington ED, Mok JM. Psychological distress in the active duty military spine patient. Mil Med. 2013;178(10):1059-1064. doi:10.7205/MILMED-D-13-00162 24083918

[11] Vasterling JJ, Duke LM, Brailey K, Constans JI, Allain AN Jr, Sutker PB. Attention, learning, and memory performances and intellectual resources in Vietnam veterans: PTSD and no disorder comparisons. Neuropsychology. 2002;16(1):5-14. doi:10.1037/0894-4105.16.1.5 11853357

[12] Vasterling JJ, Proctor SP, Amoroso P, Kane R, Heeren T, White RF. Neuropsychological outcomes of army personnel following deployment to the Iraq war. JAMA. 2006;296(5):519-529. doi:10.1001/jama.296.5.519 16882958

[13] Martindale SL, Morissette SB, Kimbrel NA, . Neuropsychological functioning, coping, and quality of life among returning war veterans. Rehabil Psychol. 2016;61(3):231-239. doi:10.1037/rep0000076 26891248 PMC5032646

[14] Vogt D, Smith B, Elwy R, . Predeployment, deployment, and postdeployment risk factors for posttraumatic stress symptomatology in female and male OEF/OIF veterans. J Abnorm Psychol. 2011;120(4):819-831. doi:10.1037/a0024457 21707125

[15] Pittman JOE, Goldsmith AA, Lemmer JA, Kilmer MT, Baker DG. Post-traumatic stress disorder, depression, and health-related quality of life in OEF/OIF veterans. Qual Life Res. 2012;21(1):99-103. doi:10.1007/s11136-011-9918-3 21516356

[16] Schneiderman AI, Braver ER, Kang HK. Understanding sequelae of injury mechanisms and mild traumatic brain injury incurred during the conflicts in Iraq and Afghanistan: persistent postconcussive symptoms and posttraumatic stress disorder. Am J Epidemiol. 2008;167(12):1446-1452. doi:10.1093/aje/kwn068 18424429

[17] Fortier CB, Amick MM, Grande L, . The Boston Assessment of Traumatic Brain Injury-Lifetime (BAT-L) semistructured interview: evidence of research utility and validity. J Head Trauma Rehabil. 2014;29(1):89-98. doi:10.1097/HTR.0b013e3182865859 23535389 PMC3997066

[18] Lindquist LK, Love HC, Elbogen EB. Traumatic brain injury in Iraq and Afghanistan veterans: new results from a national random sample study. J Neuropsychiatry Clin Neurosci. 2017;29(3):254-259. doi:10.1176/appi.neuropsych.16050100 28121256 PMC5501743

[19] O’Neil M, Carlson K, Storzbach D, . Complications of Mild Traumatic Brain Injury in Veterans and Military Personnel: A Systematic Review. Department of Veterans Affairs; 2013. 24600749

[20] Hoge CW, Castro CA, Messer SC, McGurk D, Cotting DI, Koffman RL. Combat duty in Iraq and Afghanistan, mental health problems and barriers to care. US Army Med Dep J. 2008;7-17. doi:10.1056/NEJMoa040603 20088060

[21] Chemtob CM, Muraoka MY, Wu-Holt P, Fairbank JA, Hamada RS, Keane TM. Head injury and combat-related posttraumatic stress disorder. J Nerv Ment Dis. 1998;186(11):701-708. doi:10.1097/00005053-199811000-00007 9824173

[22] Yurgil KA, Barkauskas DA, Vasterling JJ, ; Marine Resiliency Study Team. Association between traumatic brain injury and risk of posttraumatic stress disorder in active-duty Marines. JAMA Psychiatry. 2014;71(2):149-157. doi:10.1001/jamapsychiatry.2013.3080 24337530

[23] Shura RD, Nazem S, Miskey HM, ; Va Mid-Atlantic Mirecc Workgroup. Relationship between traumatic brain injury history and recent suicidal ideation in Iraq/Afghanistan-era veterans. Psychol Serv. 2019;16(2):312-320. doi:10.1037/ser0000208 30382745

[24] Taylor BC, Hagel EM, Carlson KF, . Prevalence and costs of co-occurring traumatic brain injury with and without psychiatric disturbance and pain among Afghanistan and Iraq War Veteran V.A. users. Med Care. 2012;50(4):342-346. doi:10.1097/MLR.0b013e318245a558 22228249

[25] Vanderploeg RD, Belanger HG, Curtiss G. Mild traumatic brain injury and posttraumatic stress disorder and their associations with health symptoms. Arch Phys Med Rehabil. 2009;90(7):1084-1093. doi:10.1016/j.apmr.2009.01.023 19577020

[26] Lindemer ER, Salat DH, Leritz EC, McGlinchey RE, Milberg WP. Reduced cortical thickness with increased lifetime burden of PTSD in OEF/OIF veterans and the impact of comorbid TBI. Neuroimage Clin. 2013;2:601-611. doi:10.1016/j.nicl.2013.04.009 24179811 PMC3777819

[27] Spielberg JM, McGlinchey RE, Milberg WP, Salat DH. Brain network disturbance related to posttraumatic stress and traumatic brain injury in veterans. Biol Psychiatry. 2015;78(3):210-216. doi:10.1016/j.biopsych.2015.02.013 25818631

[28] Shenton ME, Hamoda HM, Schneiderman JS, . A review of magnetic resonance imaging and diffusion tensor imaging findings in mild traumatic brain injury. Brain Imaging Behav. 2012;6(2):137-192. doi:10.1007/s11682-012-9156-5 22438191 PMC3803157

[29] Dennis EL, Disner SG, Fani N, . Altered white matter microstructural organization in posttraumatic stress disorder across 3047 adults: results from the PGC-ENIGMA PTSD consortium. Mol Psychiatry. 2019;26(8):4315-4330. doi:10.1038/s41380-019-0631-x 31857689 PMC7302988

[30] Kuo JR, Kaloupek DG, Woodward SH. Amygdala volume in combat-exposed veterans with and without posttraumatic stress disorder: a cross-sectional study. Arch Gen Psychiatry. 2012;69(10):1080-1086. doi:10.1001/archgenpsychiatry.2012.73 23026958

[31] Gurvits TV, Shenton ME, Hokama H, . Magnetic resonance imaging study of hippocampal volume in chronic, combat-related posttraumatic stress disorder. Biol Psychiatry. 1996;40(11):1091-1099. doi:10.1016/S0006-3223(96)00229-6 8931911 PMC2910907

[32] Bremner JD, Randall PR, Scott TM, . MRI-based measurement of hippocampal volume in patients with combat-related posttraumatic stress disorder. Am J Psychiatry. 1995;152(7):973-981. doi:10.1176/ajp.152.7.973 7793467 PMC3233767

[33] Aupperle RL, Connolly CG, Stillman AN, May AC, Paulus MP. Deployment and post-deployment experiences in OEF/OIF veterans: relationship to gray matter volume. PLoS One. 2013;8(9):e75880. doi:10.1371/journal.pone.0075880 24058706 PMC3776771

[34] Clausen AN, Billinger SA, Sisante JV, Suzuki H, Aupperle RL. Preliminary evidence for the impact of combat experiences on gray matter volume of the posterior insula. Front Psychol. 2017;8:2151. doi:10.3389/fpsyg.2017.02151 29312038 PMC5733022

[35] Butler O, Adolf J, Gleich T, . Military deployment correlates with smaller prefrontal gray matter volume and psychological symptoms in a subclinical population. Transl Psychiatry. 2017;7(2):e1031. doi:10.1038/tp.2016.288 28195568 PMC5438025

[36] Sizonenko SV, Camm EJ, Garbow JR, . Developmental changes and injury induced disruption of the radial organization of the cortex in the immature rat brain revealed by in vivo diffusion tensor MRI. Cereb Cortex. 2007;17(11):2609-2617. doi:10.1093/cercor/bhl168 17259644 PMC4780675

[37] Budde MD, Janes L, Gold E, Turtzo LC, Frank JA. The contribution of gliosis to diffusion tensor anisotropy and tractography following traumatic brain injury: validation in the rat using Fourier analysis of stained tissue sections. Brain. 2011;134(Pt 8):2248-2260. doi:10.1093/brain/awr161 21764818 PMC3155707

[38] Seehaus A, Roebroeck A, Bastiani M, . Histological validation of high-resolution DTI in human post mortem tissue. Front Neuroanat. 2015;9:98. doi:10.3389/fnana.2015.00098 26257612 PMC4511840

[39] Laitinen T, Sierra A, Bolkvadze T, Pitkänen A, Gröhn O. Diffusion tensor imaging detects chronic microstructural changes in white and gray matter after traumatic brain injury in rat. Front Neurosci. 2015;9:128. doi:10.3389/fnins.2015.00128 25954146 PMC4406060

[40] Bock AS, Olavarria JF, Leigland LA, Taber EN, Jespersen SN, Kroenke CD. Diffusion tensor imaging detects early cerebral cortex abnormalities in neuronal architecture induced by bilateral neonatal enucleation: an experimental model in the ferret. Front Syst Neurosci. 2010;4:149. doi:10.3389/fnsys.2010.00149 21048904 PMC2971465

[41] Dean JM, McClendon E, Hansen K, . Prenatal cerebral ischemia disrupts MRI-defined cortical microstructure through disturbances in neuronal arborization. Sci Transl Med. 2013;5(168):168ra7. doi:10.1126/scitranslmed.3004669 23325800 PMC3857141

[42] Leigland LA, Budde MD, Cornea A, Kroenke CD. Diffusion MRI of the developing cerebral cortical gray matter can be used to detect abnormalities in tissue microstructure associated with fetal ethanol exposure. Neuroimage. 2013;83:1081-1087. doi:10.1016/j.neuroimage.2013.07.068 23921100 PMC3815979

[43] Davenport ND, Lamberty GJ, Nelson NW, Lim KO, Armstrong MT, Sponheim SR. PTSD confounds detection of compromised cerebral white matter integrity in military veterans reporting a history of mild traumatic brain injury. Brain Inj. 2016;30(12):1491-1500. doi:10.1080/02699052.2016.1219057 27834537

[44] Lepage C, de Pierrefeu A, Koerte IK, . White matter abnormalities in mild traumatic brain injury with and without post-traumatic stress disorder: a subject-specific diffusion tensor imaging study. Brain Imaging Behav. 2018;12(3):870-881. doi:10.1007/s11682-017-9744-5 28676987 PMC5756136

[45] Santhanam P, Teslovich T, Wilson SH, Yeh P-H, Oakes TR, Weaver LK. Decreases in white matter integrity of ventrolimbic pathway linked to posttraumatic stress disorder in mild traumatic brain injury. J Neurotrauma. 2019;36(7):1093-1098. doi:10.1089/neu.2017.5541 30039740

[46] Lopez KC, Leary JB, Pham DL, Chou Y-Y, Dsurney J, Chan L. Brain volume, connectivity, and neuropsychological performance in mild traumatic brain injury: the impact of post-traumatic stress disorder symptoms. J Neurotrauma. 2017;34(1):16-22. doi:10.1089/neu.2015.4323 26942337 PMC5198106

[47] Gilbertson MW, Shenton ME, Ciszewski A, . Smaller hippocampal volume predicts pathologic vulnerability to psychological trauma. Nat Neurosci. 2002;5(11):1242-1247. doi:10.1038/nn958 12379862 PMC2819093

[48] McGlinchey RE, Milberg WP, Fonda JR, Fortier CB. A methodology for assessing deployment trauma and its consequences in OEF/OIF/OND veterans: the TRACTS longitudinal prospective cohort study. Int J Methods Psychiatr Res. 2017;26(3):e1556. doi:10.1002/mpr.1556 28211592 PMC5561532

[49] First MB, Gibbon M, Spitzer RL, Williams JBW. Structured Clinical Interview for DSM-IV Axis I Disorders (SCID-I). American Psychiatric Press; 1997.

[50] Blake D, Weathers F, Nagy L, . The development of a clinician-administered PTSD scale. J Trauma Stress. 1995;75-90. doi:10.1002/jts.2490080106 7712061

[51] Weathers FW, Keane TM, Davidson JR. Clinician-administered PTSD scale: a review of the first ten years of research. Depress Anxiety. 2001;13(3):132-156. doi:10.1002/da.1029 11387733

[52] King LA, King DW, Vogt DS, Knight J, Samper RE. Deployment risk and resilience inventory: a collection of measures for studying deployment-related experiences of military personnel and veterans. Mil Psychol. 2006;18(2):89-120. doi:10.1207/s15327876mp1802_1

[53] World Health Organization. World Health Organization Disability Assessment Schedule II. Accessed September 14, 2021. https://www.who.int/standards/classifications/international-classification-of-functioning-disability-and-health/who-disability-assessment-schedule

[54] Cicerone KD, Klamar K. Persistent postconcussion syndrome: the structure of subjective complaints after mild traumatic brain injury. J Head Trauma Rehabil. 1995;10(3):1-17. doi:10.1097/00001199-199510030-00002

[55] Wechsler D. Wechsler Adult Intelligence Scale (Manual). Fourth ed. Psychological Corporation; 2008.

[56] Stroop JR. Studies of interference in serial verbal reactions. J Exp Psychol. 1935;18(6):643-662. doi:10.1037/h0054651

[57] Bush G, Whalen PJ, Rosen BR, Jenike MA, McInerney SC, Rauch SL. The counting Stroop: an interference task specialized for functional neuroimaging—validation study with functional MRI. Hum Brain Mapp. 1998;6(4):270-282. doi:10.1002/(SICI)1097-0193(1998)6:4<270::AID-HBM6>3.0.CO;2-0 9704265 PMC6873370

[58] Wechsler D. WAIS-III Administration and Scoring Manual. Psychological Corporation; 1997.

[59] Morgen K, Sammer G, Courtney SM, . Evidence for a direct association between cortical atrophy and cognitive impairment in relapsing-remitting MS. Neuroimage. 2006;30(3):891-898. doi:10.1016/j.neuroimage.2005.10.032 16360321

[60] Sun X, Zhang X, Chen X, . Age-dependent brain activation during forward and backward digit recall revealed by fMRI. Neuroimage. 2005;26(1):36-47. doi:10.1016/j.neuroimage.2005.01.022 15862203

[61] Stretton J, Winston GP, Sidhu M, . Disrupted segregation of working memory networks in temporal lobe epilepsy. Neuroimage Clin. 2013;2:273-281. doi:10.1016/j.nicl.2013.01.009 24179782 PMC3777779

[62] Stretton J, Winston G, Sidhu M, . Neural correlates of working memory in temporal lobe epilepsy: an fMRI study. Neuroimage. 2012;60(3):1696-1703. doi:10.1016/j.neuroimage.2012.01.126 22330313 PMC3677092

[63] Green P. Green’s Medical Symptom Validity Test (MSVT) for Microsoft Windows User’s Manual. Green’s Publishing; 2014.

[64] Analysis Group. FMRIB Software Library v6.0. Updated August 11, 2021. Accessed August 9, 2022. https://fsl.fmrib.ox.ac.uk/fsl/fslwiki/

[65] 3D Slicer. Updated July 28, 2022. Accessed August 9, 2022. https://www.slicer.org/

[66] Free Surfer. Accessed August 9, 2022. https://surfer.nmr.mgh.harvard.edu/

[67] Destrieux C, Fischl B, Dale A, Halgren E. Automatic parcellation of human cortical gyri and sulci using standard anatomical nomenclature. Neuroimage. 2010;53(1):1-15. doi:10.1016/j.neuroimage.2010.06.010 20547229 PMC2937159

[68] Pasternak O, Sochen N, Gur Y, Intrator N, Assaf Y. Free water elimination and mapping from diffusion MRI. Magn Reson Med. 2009;62(3):717-730. doi:10.1002/mrm.22055 19623619

[69] Guenette JP, Stern RA, Tripodis Y, . Automated versus manual segmentation of brain region volumes in former football players. Neuroimage Clin. 2018;18:888-896. doi:10.1016/j.nicl.2018.03.026 29876273 PMC5988230

[70] Schumm JA, Gore WL, Chard KM, Meyer EC. Examination of the World Health Organization Disability Assessment System as a measure of disability severity among veterans receiving cognitive processing therapy. J Trauma Stress. 2017;30(6):704-709. doi:10.1002/jts.22243 29178377

[71] Stolp HB, Ball G, So PW, . Voxel-wise comparisons of cellular microstructure and diffusion-MRI in mouse hippocampus using 3D Bridging of Optically-clear histology with Neuroimaging Data (3D-BOND). Sci Rep. 2018;8(1):4011. doi:10.1038/s41598-018-22295-9 29507311 PMC5838167

[72] Blumenfeld-Katzir T, Pasternak O, Dagan M, Assaf Y. Diffusion MRI of structural brain plasticity induced by a learning and memory task. PLoS One. 2011;6(6):e20678. doi:10.1371/journal.pone.0020678 21701690 PMC3119075

[73] Sagi Y, Tavor I, Hofstetter S, Tzur-Moryosef S, Blumenfeld-Katzir T, Assaf Y. Learning in the fast lane: new insights into neuroplasticity. Neuron. 2012;73(6):1195-1203. doi:10.1016/j.neuron.2012.01.025 22445346

[74] Breu M, Reisinger D, Tao L, . In vivo high-resolution diffusion tensor imaging of the developing neonatal rat cortex and its relationship to glial and dendritic maturation. Brain Struct Funct. 2019;224(5):1815-1829. doi:10.1007/s00429-019-01878-w 31011813 PMC6565480

[75] Sofroniew MV, Vinters HV. Astrocytes: biology and pathology. Acta Neuropathol. 2010;119(1):7-35. doi:10.1007/s00401-009-0619-8 20012068 PMC2799634

[76] Palmer AL, Ousman SS. Astrocytes and aging. Front Aging Neurosci. 2018;10:337. doi:10.3389/fnagi.2018.00337 30416441 PMC6212515

[77] Boska MD, Hasan KM, Kibuule D, . Quantitative diffusion tensor imaging detects dopaminergic neuronal degeneration in a murine model of Parkinson’s disease. Neurobiol Dis. 2007;26(3):590-596. doi:10.1016/j.nbd.2007.02.010 17428671 PMC2040046

[78] Parham E, Feshki M, Soltanian-Zadeh H. Relation between brain structural connectivity and processing speed. 2018 25th National and 3rd International Iranian Conference on Biomedical Engineering. November 2018. Accessed August 11, 2022. https://www.proceedings.com/48629.html

[79] Müller MJ, Greverus D, Dellani PR, . Functional implications of hippocampal volume and diffusivity in mild cognitive impairment. Neuroimage. 2005;28(4):1033-1042. doi:10.1016/j.neuroimage.2005.06.029 16084115

[80] Anblagan D, Valdés Hernández MC, Ritchie SJ, . Coupled changes in hippocampal structure and cognitive ability in later life. Brain Behav. 2018;8(2):e00838. doi:10.1002/brb3.838 29484252 PMC5822578

[81] Marx BP, Brailey K, Proctor SP, . Association of time since deployment, combat intensity, and posttraumatic stress symptoms with neuropsychological outcomes following Iraq war deployment. Arch Gen Psychiatry. 2009;66(9):996-1004. doi:10.1001/archgenpsychiatry.2009.109 19736356

[82] Wisco BE, Pineles SL, Shipherd JC, Marx BP. Attentional interference by threat and post-traumatic stress disorder: the role of thought control strategies. Cogn Emot. 2013;27(7):1314-1325. doi:10.1080/02699931.2013.775109 23517445 PMC3748244

[83] Pineles SL, Shipherd JC, Mostoufi SM, Abramovitz SM, Yovel I. Attentional biases in PTSD: more evidence for interference. Behav Res Ther. 2009;47(12):1050-1057. doi:10.1016/j.brat.2009.08.001 19716122

[84] Ziegler DA, Janowich JR, Gazzaley A. Differential impact of interference on internally—and externally—directed attention. Sci Rep. 2018;8(1):2498. doi:10.1038/s41598-018-20498-8 29410407 PMC5802789

[85] Brown VM, Morey RA. Neural systems for cognitive and emotional processing in posttraumatic stress disorder. Front Psychol. 2012;3:449. doi:10.3389/fpsyg.2012.00449 23162499 PMC3498869

[86] Hadland KA, Rushworth MFS, Gaffan D, Passingham RE. The effect of cingulate lesions on social behaviour and emotion. Neuropsychologia. 2003;41(8):919-931. doi:10.1016/S0028-3932(02)00325-1 12667528

[87] Vasterling JJ, Aslan M, Lee LO, . Longitudinal associations among posttraumatic stress disorder symptoms, traumatic brain injury, and neurocognitive functioning in army soldiers deployed to the Iraq War. J Int Neuropsychol Soc. 2018;24(4):311-323. doi:10.1017/S1355617717001059 29199924 PMC6103787

[88] Kozlovskiy SA, Vartanov AV, Nikonova EY, Pyasik MM, Velichkovsky BM. The cingulate cortex and human memory processes. Psychol Russ State Art. 2012. doi:10.11621/pir.2012.0014

[89] Yeh P-H, Wang B, Oakes TR, . Postconcussional disorder and PTSD symptoms of military-related traumatic brain injury associated with compromised neurocircuitry. Hum Brain Mapp. 2014;35(6):2652-2673. doi:10.1002/hbm.22358 24038816 PMC6869078

[90] Misaki M, Phillips R, Zotev V, . Connectome-wide investigation of altered resting-state functional connectivity in war veterans with and without posttraumatic stress disorder. Neuroimage Clin. 2018;17:285-296. doi:10.1016/j.nicl.2017.10.032 29527476 PMC5842755

[91] Su Y-J, Chen S-H. Negative cognitions prior to trauma predict acute posttraumatic stress disorder symptomatology. J Trauma Stress. 2018;31(1):14-24. doi:10.1002/jts.22255 29513915

[92] Xue C, Ge Y, Tang B, . A meta-analysis of risk factors for combat-related PTSD among military personnel and veterans. PLoS One. 2015;10(3):e0120270. doi:10.1371/journal.pone.0120270 25793582 PMC4368749

[93] Bremner JD, Southwick SM, Johnson DR, Yehuda R, Charney DS. Childhood physical abuse and combat-related posttraumatic stress disorder in Vietnam veterans. Am J Psychiatry. 1993;150(2):235-239. doi:10.1176/ajp.150.2.235 8422073

[94] Fontana A, Rosenheck R. Posttraumatic stress disorder among Vietnam theater veterans: a causal model of etiology in a community sample. J Nerv Ment Dis. 1994;182(12):677-684. doi:10.1097/00005053-199412000-00001 7989911

[95] Davidson JR, Hughes D, Blazer DG, George LK. Post-traumatic stress disorder in the community: an epidemiological study. Psychol Med. 1991;21(3):713-721. doi:10.1017/S0033291700022352 1946860

[96] Corneil W, Beaton R, Murphy S, Johnson C, Pike K. Exposure to traumatic incidents and prevalence of posttraumatic stress symptomatology in urban firefighters in two countries. J Occup Health Psychol. 1999;4(2):131-141. doi:10.1037/1076-8998.4.2.131 10212865

[97] Luxton DD, Skopp NA, Maguen S. Gender differences in depression and PTSD symptoms following combat exposure. Depress Anxiety. 2010;27(11):1027-1033. doi:10.1002/da.20730 20721909

[98] Maguen S, Cohen B, Ren L, Bosch J, Kimerling R, Seal K. Gender differences in military sexual trauma and mental health diagnoses among Iraq and Afghanistan veterans with posttraumatic stress disorder. Womens Health Issues. 2012;22(1):e61-e66. doi:10.1016/j.whi.2011.07.010 21907590

