# Supplementary material for: Association of War Zone–Related Stress With Alterations in Limbic Gray Matter Microstructure
Source: JAMA Netw Open. 2022 Sep 16;5(9):e2231891. doi: 10.1001/jamanetworkopen.2022.31891 (PMC9482063; doi:10.1001/jamanetworkopen.2022.31891)
Supplement: Supplement. — eTable 1. Association of Limbic Gray Matter Diffusion (FAT) and Disability/Neurobehavioral Symptoms eTable 2. Association of Limbic Gray Matter Diffusion (FAT) and Hypervigilance State [file jamanetwopen-e2231891-s001.pdf]

Supplemental Online Content

Kaufmann E, Rojczyk P, Sydnor VJ, et al. Association of war zone–related stress with alterations in limbic gray matter microstructure. *JAMA Netw Open*. 2022;5(9):e2231891. doi:10.1001/jamanetworkopen.2022.31891

eTable 1. Association of Limbic Gray Matter Diffusion (FAT) and Disability/Neurobehavioral Symptoms
eTable 2. Association of Limbic Gray Matter Diffusion (FAT) and Hypervigilance State

This supplemental material has been provided by the authors to give readers additional information about their work.

© 2022 Kaufmann E et al. *JAMA Network Open.*

eTable 1 Association of Limbic Gray Matter Diffusion (FA_T) and Disability/Neurobehavioral Symptoms

		Neurobehavioral Symptom Inventory (NSI)		World Health Organization Disability Assessment Schedule II (WHODAS II)	
**Diffusion measure**	**Region**	**Partial r**	**FDR corrected p-value**	**Partial r**	**FDR corrected p-value**
FA_T	**Left amygdala-hippocampus comp.**	0.111	0.4496	0.145	0.3637
	**Left cingulate gyrus**	-0.093	0.4496	-0.116	0.3856
	**Left lateral orbitofrontal cortex**	-0.101	0.4496	-0.084	0.4598
	**Left medial orbitofrontal cortex**	-0.091	0.4496	-0.175	0.3637
	**Left parahippocampal gyrus**	-0.064	0.6743	-0.080	0.4598
	**Right amygdala-hippocampus comp.**	0.105	0.4496	0.150	0.3637
	**Right cingulate gyrus**	-0.117	0.4496	-0.110	0.3946
	**Right lateral orbitofrontal cortex**	-0.137	0.4496	-0.016	0.8416
	**Right medial orbitofrontal cortex**	-0.133	0.4496	-0.094	0.4598
	**Right parahippocampal gyrus**	-0.101	0.4496	0.072	0.4898

© 2022 Kaufmann E et al. *JAMA Network Open.*

eTable 2 Association of Limbic Gray Matter Diffusion (FA_T) and Hypervigilance State

Statistically significant results are marked with an asterisk (*). The higher the partial r, the stronger the linear relationship between two variables. Positive values represent positive, negative values represent negative or in inverse correlations. The hypervigilance state was assessed via a subscale within the clinician-administered PTSD-Scale (CAPS) including the frequency and intensity of the following symptoms: difficulty sleeping, irritability, difficulty concentrating, hypervigilance and exaggerated startle reaction.

FA_T fractional anisotropy$_{Tissue}$, FDR false discovery rate

		Hypervigilance state	
**Diffusion measure**	**Region**	**Partial r**	**FDR corrected p-value**
**FA_T**	**Left amygdala-hippocampus comp.**	0.325	<0.001*
	**Left cingulate gyrus**	-0.253	<0.01*
	**Right amygdala-hippocampus comp.**	0.309	<0.001*
	**Right cingulate gyrus**	-0.261	<0.01*

© 2022 Kaufmann E et al. *JAMA Network Open.*
